# Increased microcirculatory heterogeneity in patients with obstructive sleep apnea

**DOI:** 10.1371/journal.pone.0184291

**Published:** 2017-09-01

**Authors:** Lukas Ruzek, Karolina Svobodova, Lyle J. Olson, Ondrej Ludka, Ivan Cundrle

**Affiliations:** 1 Department of Anaesthesiology and Intensive Care, St. Anna's University Hospital Brno, Brno, Czech Republic; 2 International Clinical Research Center, St. Anna's University Hospital Brno, Brno, Czech Republic; 3 Faculty of Medicine, Masaryk University, Brno, Czech Republic; 4 Division of Cardiovascular Diseases, Mayo Clinic, Rochester, Minnesota, United States of America; 5 Internal Cardiology Department, University Hospital Brno, Brno, Czech Republic; University of Rome Tor Vergata, ITALY

## Abstract

**Introduction:**

Obstructive sleep apnea (OSA) is the most common form of sleep disordered breathing and has been associated with major cardiovascular comorbidities. We hypothesized that the microcirculation is impaired in patients with OSA and that the magnitude of impairment correlates to OSA severity.

**Methods:**

Subjects were consecutive patients scheduled for routine diagnostic polysomnography (PSG). OSA was defined by paradoxical rib cage movements together with abdominal excursions and by the apnea-hypopnea index (AHI) (events/hour; no apnea AHI<5; mild apnea 5≤AHI<15; moderate apnea 15≤AHI<30; severe apnea AHI ≥30). Sidestream darkfield imaging was used to assess the sublingual microcirculation. Recordings of sublingual microcirculation (5 random sites) were performed before and after overnight PSG. Data are summarized as mean (±SD); p values <0.05 were considered statistically significant.

**Results:**

Thirty-three consecutive patients were included. OSA was diagnosed in 16 subjects (4 moderate, 12 severe). There was no significant difference in microcirculation between subjects with moderate OSA and without OSA. However, compared to subjects without OSA, subjects with severe OSA (AHI≥30) showed a significant decrease of microvascular flow index (-0.07±0.17 vs. 0.08±0.14; p = 0.02) and increase of microvascular flow index heterogeneity (0.06±0.15 vs. -0.06±0.11; p = 0.02) overnight. Multiple regression analysis (adjusted for age and gender) showed both decrease of flow and increase of flow heterogeneity associated with AHI (b = -0.41; F = 1.8; p = 0.04 and b = 0.43; F = 1.9; p = 0.03, respectively).

**Conclusion:**

Acute overnight microcirculatory changes are observed in subjects with severe OSA characterized by decreased flow and increased flow heterogeneity.

## Introduction

Obstructive sleep apnea (OSA) is the most prevalent form of sleep disordered breathing in the community [1]. OSA is characterized by recurrent cessation of breathing during sleep and has been associated with major cardiovascular comorbidities including systemic and pulmonary hypertension, heart failure, myocardial infarction, stroke as well as increased mortality rates [2–4]. The mechanisms linking OSA and cardiovascular diseases are not fully understood [5].

Microcirculatory changes (both structural and functional changes in small arteries, arterioles and capillaries) have been suggested as a basis for end-organ damage in OSA and are important in cardiovascular disease development [6]. Indeed, multiple studies have shown different stages of endothelial dysfunction to be present in patients with OSA [7,8]. However, it has not been firmly established whether OSA microcirculatory impairment is characterized by functional or structural microvascular changes.

Sidestream dark field imaging (SDF) is a recently developed method for the clinical observation of the microcirculation [9]. SDF allows bedside, non-invasive visualization of microvascular density, flow and heterogeneity (mostly from the sublingual mucosa) [10]. To date, it has been mainly used in the intensive care setting [11]. Notably, SDF has been shown to be useful in vascular morbidity assessment [12] and to correlate with endothelial function [13].

We hypothesized that sublingual microcirculation structure and function are impaired in patients with OSA and that the degree of impairment is related to OSA severity. Furthermore, we hypothesized that microcirculatory changes are more pronounced in the morning, after awakening. Accordingly, the aim of this study was to measure sublingual microcirculatory parameters before and after diagnostic polysomnography (PSG) in subjects with OSA.

## Methods

### Subject selection

Subjects were consecutive patients scheduled for routine diagnostic PSG. Patients on treatment with continuous positive airway pressure were excluded. Informed consent was obtained from all individual participants included in the study. This study was conducted in accordance with the declaration of Helsinki and approved by the local Ethics Committee of the St. Anne's University Hospital in Brno, Czech Republic (No. 65V/2014; 08/10/2014).

### Polysomnography

All PSG studies were performed in the International Clinical Research Center sleep laboratory. PSGs were digitally recorded on an E-Series (Compumedics; Victoria, Australia) digital PSG acquisition system. Recorded parameters were four channel electroencephalogram, two-channel electrooculogram, nasal airflow and oronasal thermal sensor, submental and limb electromyograms, and three channel ECG, transcutaneous pulse oximetry, thoracic and abdominal inductance plethysmography, snore sensor, and body-position sensor.

All PSG studies were scored for sleep stages and disordered breathing events according to the American Academy of Sleep Medicine scoring guidelines [14] by board registered PSG sleep technologists. As per published guidelines [14], sleep apnea was defined by the type (central/obstructive/mixed apnea) and by the apnea-hypopnea index (AHI) (events/hour; no apnea AHI<5; mild apnea 5≤AHI<15; moderate apnea 15≤AHI<30; severe apnea AHI ≥30). Apneas were defined as ≥ 90% reduction of airflow for at least 10 seconds. Obstructive apnea was defined by paradoxical rib cage movements together with abdominal excursions; central apnea was defined by the absence of thoracic and abdominal wall movements. Mixed apnea was defined by absent inspiratory effort in the initial part of the event and resumption of inspiratory effort in the second part of the event. Hypopneas were defined as a reduction in nasal pressure of ≥ 30% of the baseline for at least 10 seconds together with oxygen (O_2_) desaturation of at least 3% from the pre-event baseline or with an arousal.

### Sidestream darkfield imaging (SDF)

**Fig 1:**
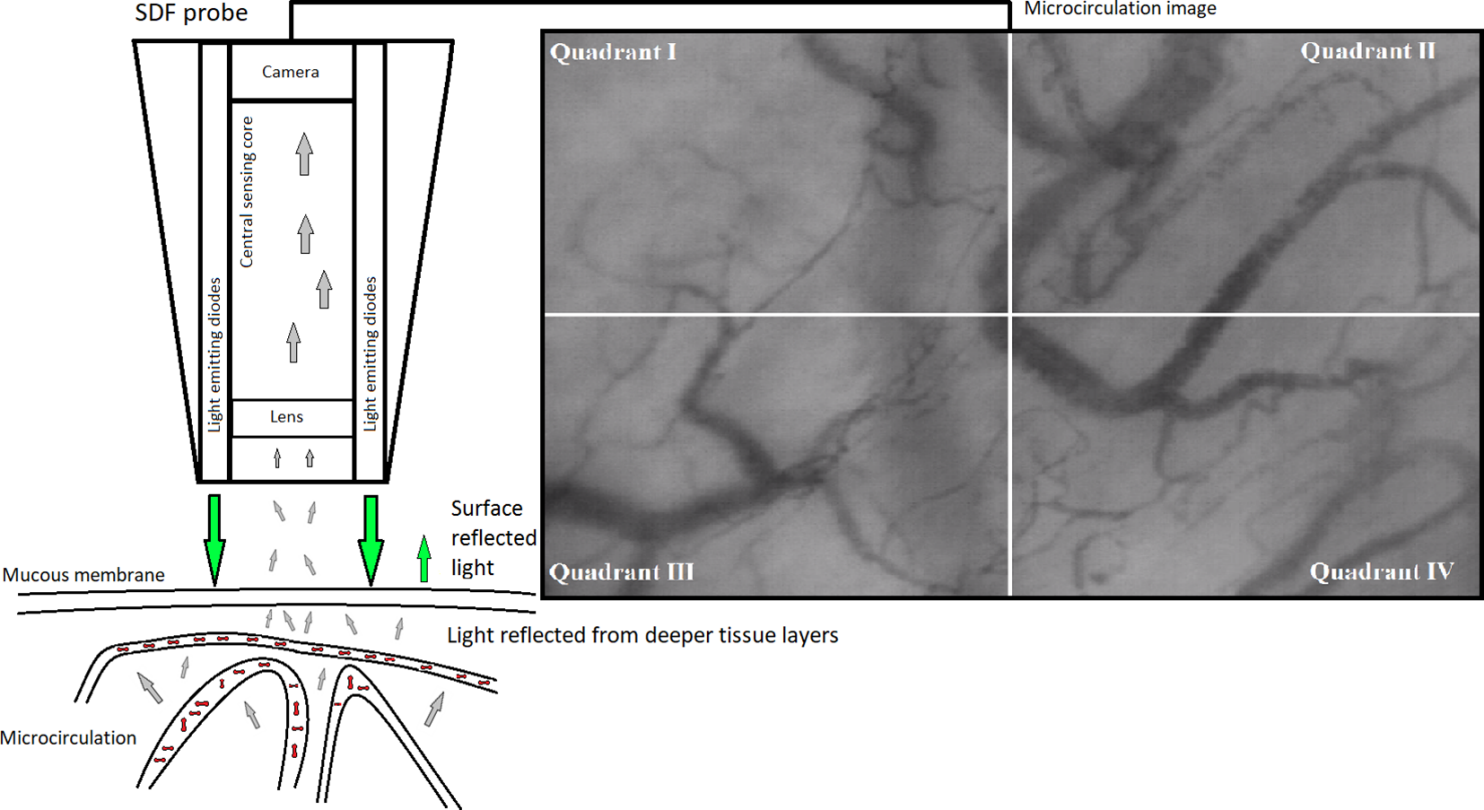
Sidestream darkfield imaging. Sidestream darkfield imaging scheme with an example of microvascular flow index (MFI) measurement. MFI is based on determination of predominant type of flow in four quadrants; flow is characterized as absent (0), intermittent (1), sluggish (2), continuous (3) and hyperdynamic (4). The values of the four analyzed quadrants are then averaged to create MFI.

SDF imaging (MicroScan Video Microscope, MicroVisionMedical, Inc) was used for the monitoring of microcirculation before and after PSG. SDF uses light emitting diodes (green light of a 530 nm wavelength, which is absorbed by erythrocytes) placed around sensing central probe to illuminate tissue microcirculation. Optical separation of emitting diodes and sensing core prevents surface reflected light to interfere with the image of microcirculation. Only light reflected form deeper tissue layers may enter the central sensing probe and create microcirculation image (Fig 1) [15]. SDF measurements were scheduled for 6:00 am and 9:00 pm. Before each measurement, subjects were allowed to rest 30 minutes in a supine position and were allowed to drink a glass of water to clear saliva from the oral cavity. SDF video recordings were taken only after obtaining sharp images without signs of pressure artifacts as recommended previously [10]. Five video sequences (20 s each) were taken at different random sites of the sublingual mucosa.

Video sequences were analyzed blindly off-line using software AVA 3.0 (AMS, Amsterdam, NL) by one investigator. The following parameters were analyzed as previously recommended [10]: total vessel density (TVD), perfused vessel density (PVD), microvascular flow index (MFI) and De Backer score. In brief, TVD is a density parameter that shows how many millimeters of vessels smaller than 20 μm can be found in one mm^2^ of target tissue. MFI is a flow parameter based on determination of predominant type of flow in four quadrants; flow is characterized as absent (0), intermittent (1), sluggish (2), continuous (3) and hyperdynamic (4). PVD (combination of flow and density parameter) is an estimate of functional capillary density (density of vessels with at least sluggish flow). The values of the four analyzed quadrants were averaged. DeBacker score describes capillary heterogeneity in one analyzed site; it is defined by the number of crossing capillaries in a 3x3 grid, created during video analysis. For all measured parameters, values from all 5 recorded sites were then averaged and analyzed. Finally, heterogeneity indices were tabulated for both TVD and MFI to evaluate heterogeneity in each of 5 recorded videos. The heterogeneity index was calculated as previously described: the highest minus the lowest value, divided by the mean value of measured sites [10].

### Statistics

The Shapiro-Wilkov test was used to test data for normality. One-way analysis of variance followed by post-hoc Tukey HSD test (for continuous variables); or Chi-square test (for categorical variables) were used to test for differences among the groups (no OSA/moderate OSA/severe OSA). Pearson correlation coefficient was used to test statistical dependence of microvascular parameters significantly different among groups and PSG parameters. Two multiple regression analysis models (adjusted for sex and age) were then created to assess the relationship between 1) decrease in flow (ΔMFI) and 2) increase in flow heterogeneity (ΔMFI _heterogeneity_) and AHI. The results were expressed as the beta coefficient, F ratio and p value. Data are summarized as mean (±SD) or median (IQR) where appropriate; p values <0.05 were considered statistically significant. Statistical analysis was performed using Statistica 12.0 (StatSoft Inc., Prague, Czech Republic).

## Results

Thirty-three consecutive patients were included, from whom 320 video sequences were analyzed. Subject characteristics are shown in **Table 1**. Compared to subjects without OSA, subjects with severe OSA had significantly higher BMI. Otherwise there were no significant differences in age, number of males, number of smokers, morning time to SDF measurements, frequency of major chronic illnesses, or cardiovascular medication between the groups.

**Table 1:** Subject characteristics and PSG results.

	no OSA (n = 17)	moderate OSA (n = 4)	severe OSA (n = 12)	ANOVA or χ^2^ p
Male No. (%)	14 (82)	4 (100)	12 (100)	0.21
Age (years)	51 ± 12	58 ± 16	54 ± 9	0.49
BMI (kg/m^2^)	30 ± 6	30 ± 2	36 ± 6 *	0.02
Awakening to SDF (min)	43 ± 18	32 ± 18	42 ± 21	0.55
Major Comorbidities				
Hypertension No. (%)	10 (60)	3 (75)	8 (67)	0.80
CHD No. (%)	5 (29)	0	3 (25)	0.47
Diabetes mellitus No. (%)	3 (18)	2 (50)	1 (8)	0.17
Chronic Limb Ischemia No. (%)	2 (12)	0	1 (8)	0.76
COPD No. (%)	2 (12)	0	2 (17)	0.67
Medication				
Beta blockers No. (%)	8 (47)	3 (75)	5 (42)	0.51
ACE-I or ARB No. (%)	5 (29)	3 (75)	3 (25)	0.16
Calcium channel blockers No. (%)	2 (12)	0	2 (17)	0.67
Diuretics No. (%)	2 (12)	1 (25)	3 (25)	0.62
Smoker	8 (47)	1 (25)	7 (58)	0.51

data shown as mean ± SD

* indicates significant difference compared to no OSA

χ^2^ = chi-squared test; ACE = angiotensin converting enzyme inhibitor; ARB = angiotensin II receptor blocker; BMI = body mass index; CHD = coronary heart disease; COPD = chronic obstructive pulmonary disease; OSA = obstructive sleep apnea

Polysomnography results are shown in **Table 2**. Subjects with severe OSA had significantly longer stage 1 non-rapid eye movement (NREM) sleep, shorter stage 3 NREM sleep and shorter rapid eye movement (REM) sleep compared to subjects without OSA. Furthermore, the number of obstructive apneas (events/hour) and AHI was higher and minimal O_2_ saturation was lower in subjects with severe OSA compared to subjects with moderate or without OSA. Compared to subjects without OSA, REM sleep was significantly shorter also in subjects with moderate OSA.

**Table 2:** Polysomnography results.

	no OSA (n = 17)	moderate OSA (n = 4)	severe OSA (n = 12)	ANOVA or χ^2^ p
Total sleep time (min)	361 (291–402)	298 (224–347)	311 (256–398)	0.60
NREM 1 (%)	17 (9–25)	38 (23–50)	45 (40–56)*	<0.01
NREM 2 (%)	48 (42–53)	32 (29–41)	39 (38–49)	0.17
NREM 3 (%)	15 (10–24)	17 (7–33)	2 (0–11)*§	<0.01
REM (%)	18 ± 7	9 ± 8*	6 ± 3*	<0.01
CA (events/hour)	0.7 (0–3.5)	0 (0–0)	0.4 (0–1.7)	0.56
OA (events/hour)	0.9 (0–2.9)	2 (0.6–3.7)	45.3 (21.5–63.9)* §	<0.01
Mix apnea (events/hour)	0 (0–1.0)	0 (0–0)	1.1 (0.1–5.8)	0.70
Hypopnea (events/hour)	12.4 (6.5–22)	16.4 (14.3–21.6)	17.5 (15.1–21.0)	0.44
AHI (events/hour)	15.2 (9.4–33.2)	17.3 (16.6–23.7)	64.5 (48.4–91.3)* §	<0.01
mean O_2_ saturation	95 (93–95)	95 (92–96)	92 (87–94)	0.07
minimal O_2_ saturation	85 (82–87)	84 (76–89)	67 (58–77)* §	<0.01

data shown as mean ± SD or median (IQR)

* indicates significant difference compared to no OSA

§ indicates significant difference compared to moderate OSA

χ^2^ = chi-squared test; AHI = apnea-hypopnea index; CA = central apnea; OA = obstructive apnea; NREM = non-rapid eye movement sleep; REM = rapid eye movement sleep; OSA = obstructive sleep apnea

Results of comparison of SDF microcirculatory parameters are shown in **Table 3**. There were no significant differences in the microcirculatory parameters between subjects with moderate OSA and without OSA. However, when compared to subjects without OSA, microcirculatory changes were seen in subjects with severe OSA (AHI≥30) for whom there was a significant decrease in flow (ΔMFI) and increase in flow heterogeneity (ΔMFI _heterogeneity_) during the night. Both ΔMFI and ΔMFI _heterogeneity_ correlated with AHI (r = -0.38; p = 0.03 and r = 0.41; p = 0.02, respectively). Multiple regression analysis (adjusted for age and gender) showed both decrease of flow and increase of flow heterogeneity remained significantly correlated to AHI (b = -0.41; F = 1.8; p = 0.04 and b = 0.43; F = 1.9; p = 0.03, respectively).

**Table 3:** Comparison of microcirculatory parameters in subjects with and without OSA.

	no OSA (n = 17)	moderate OSA (n = 4)	severe OSA (n = 12)	ANOVA or χ^2^ p
before PSG (evening)				
TVD (mm/mm^2^)	8.2 ± 1.1	8.6 ± 0.7	8.5 ± 0.8	0.68
PVD (mm/mm^2^)	8.2 ± 1.1	8.6 ± 0.7	8.4 ± 0.9	0.75
MFI	2.8 (2.7–3.0)	2.7 (2.6–2.9)	2.9 (2.8–3.0)	0.56
De Backer (1/mm)	5.2 ± 0.8	5.6 ± 0.8	5.5 ± 0.5	0.39
TVD _heterogeneity_	0.2 ± 0.1	0.2 ± 0.1	0.2 ± 0.1	0.42
MFI _heterogeneity_	0.2 ± 0.1	0.1 ± 0.1	0.1 ± 0.1	0.63
after PSG (morning)				
TVD (mm/mm^2^)	8.2 ± 1.5	9.5 ± 1.1	8.8 ± 0.8	0.17
PVD (mm/mm^2^)	8.2 ± 1.5	9.5 ± 1.0	8.8 ± 0.8	0.18
MFI	2.9 (2.8–3.0)	2.9 (2.8–3.0)	2.8 (2.6–3.0)	0.17
De Backer (1/mm)	5.2 ± 0.8	6.3 ± 0.6	5.5 ± 0.8	0.05
TVD _heterogeneity_	0.3 (0.2–0.4)	0.1 (0.1–0.2)	0.2 (0.2–0.3)	0.16
MFI _heterogeneity_	0.1 (0–0.1)	0.1 (0–0.2)	0.2 (0.1–0.3)	0.20
delta (morning—evening)				
Δ TVD	-0.42 (-0.66–0.48)	0.77 (-0.23–2.11)	0.27 (-0.31–0.86)	0.27
Δ PVD	-0.39 (-0.66–0.48)	0.77 (-0.23–2.09)	0.29 (-0.36–0.93)	0.28
Δ MFI	0.08 ± 0.14	0.14 ± 0.10	-0.07 ± 0.17 *	0.02
Δ De Backer	-0.05 ± 0.60	0.74 ± 1.26	0.04 ± 0.84	0.22
Δ TVD _heterogeneity_	0.07 ± 0.17	- 0.03 ± 0.05	0.01 ± 0.15	0.39
Δ MFI _heterogeneity_	-0.06 ± 0.11	- 0.06 ± 0.06	0.06 ± 0.15 *	0.05

data shown as mean ± SD or median (IQR)

* indicates significant difference compared to no OSA

χ^2^ = chi-squared test; Δ = delta; MFI = microvascular flow index; OSA = obstructive sleep apnea; PSG = polysomnography; PVD = perfused vessel density; TVD = total vessel density.

## Discussion

The major finding of this pilot study was that microcirculatory changes were present only in subjects with severe OSA and were characterized by an overnight decrease of flow and increase of flow heterogeneity.

In our study, morning to evening changes in microcirculatory flow and its heterogeneity were impaired in subjects with severe OSA. This finding is in agreement with prior studies demonstrating endothelial dysfunction (measured by flow mediated vasodilatation) in patients with OSA [7,8]. Moreover, impaired myocardial tissue perfusion has been previously demonstrated in patients with OSA and myocardial infarction [16] and an improvement of myocardial perfusion reserve has been demonstrated in OSA patients on CPAP treatment [17]. In contrast, we did not observe significant changes in microvascular density in patients with OSA. Whether microvascular density is impaired in OSA patients is not clear. Increased muscle capillary density has been previously shown in patients with OSA [18] and may be related to chronic hypoxia, which promotes angiogenesis [19]. Indeed, an insignificant trend towards higher capillary density was present in our subjects with OSA. On the other hand, some studies have shown lower capillary density in patients with OSA [20,21]. The discrepancies between prior studies and our observations may be attributed to different sites (vascular beds) and different techniques used for microcirculatory measurement.

In our subjects, flow impairment was detected only in those with severe OSA. Whether mild OSA is associated with endothelial dysfunction is controversial. Two prior studies have shown endothelial dysfunction in patients with mild OSA [22,23] whereas Blomster et al. showed no endothelial dysfunction in patients with mild OSA [24]. Time from awakening to SDF measurement did not differ between subjects with severe OSA and other subjects, suggesting the observed microcirculatory changes were not caused by different time of measurement. It is also in agreement with prior findings showing OSA changes may persist after awakening [25].

The observed microcirculatory changes were small, despite high OSA severity (median AHI 64.5). Catecholamine levels were found to be related to OSA severity [26]. Dose dependent vasoconstrictive effect of both epinephrine and norepinephrine is known [27]. Therefore, more pronounced microvascular changes may be expected in patients with severe OSA (higher catecholamine levels). However, a reduced vascular response to alpha and beta catecholamine receptor stimulation was also found in patients with severe OSA, suggesting a down-regulation of vascular receptors [28], which may have accounted for the only minor microvascular flow changes observed in our severe OSA subjects.

Although the observed microcirculatory changes were small, increases of microvascular heterogeneity during the night in patients with severe OSA may be important, as increased flow heterogeneity may prolong O_2_ diffusion distances, creating areas of tissue hypoxia in the most vulnerable sites of tissue (“lethal corner” sites most distant from all capillaries) [29] and thereby promoting organ injury.

In contrast to subjects with severe OSA, no OSA subjects tended to improve microcirculatory flow parameters (Δ MFI and Δ MFI _heterogeneity_) during the night (Table 3). We speculate the observed slight increase of microvascular flow and decrease of flow heterogeneity may have been caused by circadian changes in sympathetic nervous system tone. In contrast to patients with OSA, whose sympathetic nervous system is over-activated during the night [30], healthy subjects exhibit a circadian decrease of both sympathoadrenal and noradrenergic branch activity during the night [31]. Furthermore, sympathetic nervous system activation seems to correlate with OSA severity [32], which may explain that microcirculatory impairment occurred only in subjects with severe OSA. Moreover, both sympathetic nervous system branch activities are down-regulated during REM sleep [31], which is shorter in patients with OSA [33], and was also shorter in our subject cohort (Table 2). Indeed, the decrease of microvascular flow significantly positively correlated with the length of REM sleep (r = 0.38; p = 0.04).

### Limitations

Our study had several important limitations. First, our study cohort was small; further studies with greater numbers of subjects are necessary to confirm our findings. Second, there were only 4 subjects diagnosed with moderate OSA, which limits our findings.

Sidestream dark-field imaging is very sensitive for pressure artifacts, movement artifacts and the video sequence analysis is only partially automated, making it semi-quantitative. To mitigate this, great care was taken to obtain sharp images without pressure or movement artifacts as recommended previously [10] and video sequences were analyzed by a single skilled investigator. Despite this, in two subjects, morning video sequences were not analyzed because of poor technical quality.

It is still a matter of debate whether changes in sublingual microcirculation may be used as a surrogate for other vascular beds. Sublingual microcirculation has been suggested as a good surrogate for splanchnic blood flow [34] and indeed a low capillary supply and muscle dysfunction of palate muscles has been observed in patients with OSA and have been suggested as not only a consequence of OSA, but also as a possible contributor to worsening upper airway obstruction [35]. Furthermore, sublingual microvascular impairment was shown to be a good predictor of coronary artery disease [12] and MFI was shown to correlate with endothelial function [13]. Additionally, sublingual microcirculation has also been shown to reflect microvascular perfusion of the eye [36] and to be impaired in pulmonary hypertension [37] suggesting changes in the sublingual microcirculation may also reflect changes of other vascular beds.

## Conclusion

Microcirculatory changes are observed only in subjects with severe OSA and are characterized by overnight decreased flow and increased flow heterogeneity.

## Supporting information

S1 Table Dataset. Minimal dataset.
(XLSX)

## References

[1] Hiestand DM, Britz P, Goldman M, Phillips B. Prevalence of symptoms and risk of sleep apnea in the US population: Results from the national sleep foundation sleep in America 2005 poll. Chest. 2006;130: 780–786. doi: 10.1378/chest.130.3.780 16963675 10.1378/chest.130.3.780

[2] Marshall NS, Wong KKH, Liu PY, Cullen SRJ, Knuiman MW, Grunstein RR. Sleep Apnea as an Independent Risk Factor for All-Cause Mortality: The Busselton Health Study. Sleep. 2008;31: 1079–1085. 18714779 PMC2542953

[3] Partinen M, Jamieson A, Guilleminault C. Long-term outcome for obstructive sleep apnea syndrome patients. Mortality. Chest. 1988;94: 1200–1204. 3191760 10.1378/chest.94.6.1200

[4] Young T, Finn L, Peppard PE, Szklo-Coxe M, Austin D, Nieto FJ, et al Sleep Disordered Breathing and Mortality: Eighteen-Year Follow-up of the Wisconsin Sleep Cohort. Sleep. 2008;31: 1071–1078. 18714778 PMC2542952

[5] Wiernsperger N, Nivoit P, Bouskela E. Obstructive sleep apnea and insulin resistance: a role for microcirculation? Clinics. 2006;61: 253–266. doi: /S1807-59322006000300011 16832559 10.1590/s1807-59322006000300011

[6] Struijker-Boudier HAJ, Heijnen BFJ, Liu Y-P, Staessen JA. Phenotyping the Microcirculation. Hypertension. 2012;60: 523–527. doi: 10.1161/HYPERTENSIONAHA.111.188482 22733471 10.1161/HYPERTENSIONAHA.111.188482

[7] Patt BT, Jarjoura D, Haddad DN, Sen CK, Roy S, Flavahan NA, et al Endothelial dysfunction in the microcirculation of patients with obstructive sleep apnea. Am J Respir Crit Care Med. 2010;182: 1540–1545. doi: 10.1164/rccm.201002-0162OC 20656942 10.1164/rccm.201002-0162OC PMC3029939

[8] Kato M, Roberts-Thomson P, Phillips BG, Haynes WG, Winnicki M, Accurso V, et al Impairment of endothelium-dependent vasodilation of resistance vessels in patients with obstructive sleep apnea. Circulation. 2000;102: 2607–2610. 11085964 10.1161/01.cir.102.21.2607

[9] Goedhart PT, Khalilzada M, Bezemer R, Merza J, Ince C. Sidestream Dark Field (SDF) imaging: a novel stroboscopic LED ring-based imaging modality for clinical assessment of the microcirculation. Opt Express. 2007;15: 15101–15114. 19550794 10.1364/oe.15.015101

[10] De Backer D, Hollenberg S, Boerma C, Goedhart P, Büchele G, Ospina-Tascon G, et al How to evaluate the microcirculation: report of a round table conference. Crit Care. 2007;11: R101 doi: 10.1186/cc6118 17845716 10.1186/cc6118 PMC2556744

[11] Bezemer R, Bartels SA, Bakker J, Ince C. Clinical review: Clinical imaging of the sublingual microcirculation in the critically ill—where do we stand? Crit Care. 2012;16: 224 doi: 10.1186/cc11236 22713365 10.1186/cc11236 PMC3580600

[12] Djaberi R, Schuijf JD, Koning EJ de, Wijewickrama DC, Pereira AM, Smit JW, et al Non-invasive assessment of microcirculation by sidestream dark field imaging as a marker of coronary artery disease in diabetes. Diabetes and Vascular Disease Research. 2013;10: 123–134. doi: 10.1177/1479164112446302 22621919 10.1177/1479164112446302

[13] Yacoub S, Lam PK, Vu LHM, Le TL, Ha NT, Toan TT, et al Association of Microvascular Function and Endothelial Biomarkers With Clinical Outcome in Dengue: An Observational Study. J Infect Dis. 2016;214: 697–706. doi: 10.1093/infdis/jiw220 27230099 10.1093/infdis/jiw220 PMC4978369

[14] Berry RB, Budhiraja R, Gottlieb DJ, Gozal D, Iber C, Kapur VK, et al Rules for scoring respiratory events in sleep: update of the 2007 AASM Manual for the Scoring of Sleep and Associated Events. Deliberations of the Sleep Apnea Definitions Task Force of the American Academy of Sleep Medicine. J Clin Sleep Med. 2012;8: 597–619. doi: 10.5664/jcsm.2172 23066376 10.5664/jcsm.2172 PMC3459210

[15] Ince C. Sidestream dark field imaging: an improved technique to observe sublingual microcirculation. Crit Care. 2005;9: P72 doi: 10.1186/cc3135

[16] Nakashima H, Muto S, Amenomori K, Shiraishi Y, Nunohiro T, Suzuki S. Impact of obstructive sleep apnea on myocardial tissue perfusion in patients with ST-segment elevation myocardial infarction. Circ J. 2011;75: 890–896. 21301132 10.1253/circj.cj-10-0768

[17] Nguyen PK, Katikireddy CK, McConnell MV, Kushida C, Yang PC. Nasal continuous positive airway pressure improves myocardial perfusion reserve and endothelial-dependent vasodilation in patients with obstructive sleep apnea. J Cardiovasc Magn Reson. 2010;12: 50 doi: 10.1186/1532-429X-12-50 20815898 10.1186/1532-429X-12-50 PMC2945335

[18] Wåhlin Larsson B, Kadi F, Ulfberg J, Piehl Aulin K. Skeletal muscle morphology and aerobic capacity in patients with obstructive sleep apnoea syndrome. Respiration. 2008;76: 21–27. doi: 10.1159/000126492 18408358 10.1159/000126492

[19] Deveci D, Marshall JM, Egginton S. Chronic hypoxia induces prolonged angiogenesis in skeletal muscles of rat. Exp Physiol. 2002;87: 287–291. 12089595 10.1113/eph8702377

[20] Stål PS, Lindman R, Johansson B. Capillary supply of the soft palate muscles is reduced in long-term habitual snorers. Respiration. 2009;77: 303–310. doi: 10.1159/000197975 19176947 10.1159/000197975

[21] Nazzaro P, Schirosi G, Clemente R, Battista L, Serio G, Boniello E, et al Severe obstructive sleep apnoea exacerbates the microvascular impairment in very mild hypertensives. Eur J Clin Invest. 2008;38: 766–773. doi: 10.1111/j.1365-2362.2008.02011.x 18837802 10.1111/j.1365-2362.2008.02011.x

[22] Ciccone MM, Favale S, Scicchitano P, Mangini F, Mitacchione G, Gadaleta F, et al Reversibility of the endothelial dysfunction after CPAP therapy in OSAS patients. Int J Cardiol. 2012;158: 383–386. doi: 10.1016/j.ijcard.2011.01.065 21353713 10.1016/j.ijcard.2011.01.065

[23] Kohler M, Craig S, Pepperell JCT, Nicoll D, Bratton DJ, Nunn AJ, et al CPAP improves endothelial function in patients with minimally symptomatic OSA: results from a subset study of the MOSAIC trial. Chest. 2013;144: 896–902. doi: 10.1378/chest.13-0179 23702567 10.1378/chest.13-0179

[24] Blomster H, Laitinen T, Lyyra-Laitinen T, Vanninen E, Gylling H, Peltonen M, et al Endothelial function is well preserved in obese patients with mild obstructive sleep apnea. Sleep Breath. 2014;18: 177–186. doi: 10.1007/s11325-013-0867-7 23733256 10.1007/s11325-013-0867-7

[25] Kasai T, Bradley TD. Obstructive sleep apnea and heart failure: pathophysiologic and therapeutic implications. J Am Coll Cardiol. 2011;57: 119–127. doi: 10.1016/j.jacc.2010.08.627 21211682 10.1016/j.jacc.2010.08.627

[26] Vardhan V, Shanmuganandan K. Hypertension and catecholamine levels in sleep apnoea. Med J Armed Forces India. 2012;68: 33–38. doi: 10.1016/S0377-1237(11)60128-7 24669036 10.1016/S0377-1237(11)60128-7 PMC3862558

[27] Škorjanc A, Belušič G. Investigation of blood flow and the effect of vasoactive substances in cutaneous blood vessels of *Xenopus laevis*. Advances in Physiology Education. 2015;39: 91–95. doi: 10.1152/advan.00160.2014 26031724 10.1152/advan.00160.2014

[28] Grote L, Kraiczi H, Hedner J. Reduced α—and β2-Adrenergic Vascular Response in Patients with Obstructive Sleep Apnea. Am J Respir Crit Care Med. 2000;162: 1480–1487. doi: 10.1164/ajrccm.162.4.9912028 11029365 10.1164/ajrccm.162.4.9912028

[29] Vincent PJ-L. Yearbook of Intensive Care and Emergency Medicine 2001 Springer Science & Business Media; 2013.

[30] Bisogni V, Pengo MF, Maiolino G, Rossi GP. The sympathetic nervous system and catecholamines metabolism in obstructive sleep apnoea. J Thorac Dis. 2016;8: 243–254. doi: 10.3978/j.issn.2072-1439.2015.11.14 26904265 10.3978/j.issn.2072-1439.2015.11.14 PMC4739957

[31] Dodt C, Breckling U, Derad I, Fehm HL, Born J. Plasma epinephrine and norepinephrine concentrations of healthy humans associated with nighttime sleep and morning arousal. Hypertension. 1997;30: 71–76. 9231823 10.1161/01.hyp.30.1.71

[32] Lam JCM, Yan CSW, Lai AYK, Tam S, Fong DYT, Lam B, et al Determinants of daytime blood pressure in relation to obstructive sleep apnea in men. Lung. 2009;187: 291–298. doi: 10.1007/s00408-009-9161-7 19653037 10.1007/s00408-009-9161-7

[33] Mahmood K, Akhter N, Eldeirawi K, Önal E, Christman JW, Carley DW, et al Prevalence of Type 2 Diabetes in Patients with Obstructive Sleep Apnea in a Multi-Ethnic Sample. J Clin Sleep Med. 2009;5: 215–221. 19960641 PMC2699165

[34] Donati A, Domizi R, Damiani E, Adrario E, Pelaia P, Ince C, et al From Macrohemodynamic to the Microcirculation, From Macrohemodynamic to the Microcirculation. Critical Care Research and Practice, Critical Care Research and Practice. 2013;2013, 2013: e892710. doi: 10.1155/2013/892710 23509621 10.1155/2013/892710 PMC3600213

[35] Stål PS, Johansson B. Abnormal Mitochondria Organization and Oxidative Activity in the Palate Muscles of Long-Term Snorers with Obstructive Sleep Apnea. Respiration. 2012;83: 407–417. doi: 10.1159/000336040 22378021 10.1159/000336040

[36] Pranskunas A, Rasimaviciute R, Milieskaite E, Vitkauskiene A, Dobozinskas P, Veikutis V, et al Early course of microcirculatory perfusion in the eye and digestive tract during experimental sepsis. Crit Care. 2012;16: P203 doi: 10.1186/cc10810 10.1186/cc11341 PMC3580626 22587828

[37] Dababneh L, Cikach F, Alkukhun L, Dweik RA, Tonelli AR. Sublingual microcirculation in pulmonary arterial hypertension. Ann Am Thorac Soc. 2014;11: 504–512. doi: 10.1513/AnnalsATS.201308-277OC 24601682 10.1513/AnnalsATS.201308-277OC PMC4225801

